# DFCE-KanT: predicting spatial gene expression from histology images via contrastive learning

**DOI:** 10.1093/bioinformatics/btag502

**Published:** 2026-07-08

**Authors:** Fang Li, Pengyu Wang, Junjie Shen, Yazhou Wu

**Affiliations:** Department of Health Statistics, College of Preventive Medicine, Army Medical University, Chongqing, 400038, China; Department of Health Statistics, College of Preventive Medicine, Army Medical University, Chongqing, 400038, China; Department of Health Statistics, College of Preventive Medicine, Army Medical University, Chongqing, 400038, China; Center for Medical Big Data and Artificial Intelligence, Southwest Hospital, the First Affiliated Hospital of the Army Military Medical University, Chongqing, 400038, China; Department of Health Statistics, College of Preventive Medicine, Army Medical University, Chongqing, 400038, China

## Abstract

**Motivation:**

Spatial transcriptomics (ST) can quantitatively characterize the spatial molecular profile in tissues and yet is costly to be applied at a large scale. One viable, a low-cost alternative would be to directly predict spatial gene expression from routine hematoxylin eosin (H&E) stained WSIs. Yet, the spatial context of the H&E image corresponding to a ST is not effectively utilized in current deep learning approaches.

**Results:**

We present DFCE-KanT, which is a contrastive learning framework combining the tissue image features, gene features, and spatial location information for predicting spatial gene expressions from HE images. DenseNet incorporated a Feature Channel Enhancement (FCE) attention technique to extract features from the H&E images. A KanT, which is composed of a Kolmogorov-Arnold Network (KAN) and Transformer multi-head attention that can learn the best fusion strategy automatically in terms of fusing space information via positional encoding, increasing the ability to capture complex data patterns. This structure enhances the nonlinear ability for the projection head, modeling complex interaction between features and promoting the compatibility between image and gene expression features in the common embedding space. Experiments on five publicly available datasets (HER2+, cSCC, Alex, HBC, and Liver) demonstrate that DFCE-KanT performs noticeably better than existing methods, validating its efficacy in spatial gene expression prediction.

**Availability:**

The source code and data are available at GitHub (https://github.com/LFfocus/DFCE-KanT) and Zenodo (https://zenodo.org/records/20637079).

## 1 Introduction

The spatial distribution patterns of gene expression within tissues are pivotal for deciphering tissue architecture, functional heterogeneity, and disease mechanisms (Rao *et al.* 2021, Tian *et al.* 2023, Valdeolivas *et al.* 2024). Traditional transcriptomics methods like bulk RNA sequencing, allow for the quantification of gene expression, yet lose spatial information. While single-cell RNA sequencing addresses cell heterogeneity, also lacks of any spatial information (Rosenberg *et al.* 2018, Alon *et al.* 2021, Chen *et al.* 2022). Breakthroughs in Spatial Transcriptomics (ST) technology now allow high-throughput measurement of gene expression while preserving spatial coordinates, providing unprecedented view of the tissue microenvironment (Ståhl *et al.* 2016, Zuo *et al.* 2025). However, ST requires expensive in situ sequencing and dedicated instrumentation, severely limits its applicability to large scale clinical trials (Marx 2021, Alexandrov *et al.* 2023, Saul and Kosinsky 2023). In contrast, hematoxylin and eosin (H&E)-stained whole slide images (WSIs) have obvious advantages in terms of low-cost and high-accessibility (Crosetto *et al.* 2015, Moor and Itzkovitz 2017, Waylen *et al.* 2020). Numerous studies have verified that gene expression patterns and histological images are strongly correlated (Mondol *et al.* 2023, Tsai *et al.* 2023). Consequently, developing algorithms that can predict spatial gene expression from H&E images, is now an important avenue of research in overcoming the cost barrier for ST technology.

In recent years, numerous deep learning-based methods have been developed to establish mapping relationships between H&E images and spatial gene expression, offering a faster and more economical alternative to expensive ST technology (He *et al.* 2020, Shmatko *et al.* 2022, Wang *et al.* 2025). Representative works include ST-Net (He *et al.* 2020), which pioneered this field by using DenseNet (Simonyan and Zisserman 2015) to extract image features and mapping them to the gene dimension via fully connected layers. HisToGene (Pang *et al.* 2021), which employs a Vision Transformer (Dosovitskiy *et al.* 2021) (ViT) to capture global features through self-attention before projection. His2ST (Zeng *et al.* 2022), which integrates convolutional operations, Transformers, and graph neural networks (Kipf and Welling 2017) to extract intrinsic neighborhood relationships, with predictions based on a zero-inflated negative binomial distribution (Eraslan *et al.* 2019). THItoGene (Jia *et al.* 2024), which leverages dynamic convolution and capsule networks to mine latent molecular signals. Among them, ST-Net relies solely on convolutional operations to extract features from image patches without incorporating spatial information. HisToGene improves somewhat by modeling spatial relationships between spots. Hist2ST and THItoGene achieve further gains through enhanced image feature extraction and local point relationship modeling, but they still neglect inter-slice heterogeneity and intra-slice complexity. In addition, BLEEP (Xie *et al.* 2024) and mclSTExp (Min *et al.* 2024) apply contrastive learning to align image and gene expression features in a joint embedding space, but still exhibit suboptimal performance.

While these methods have made significant advancements in modeling the H&E-spatial gene expression association, they still face limitations: (i) High cellular density, staining batch variations, and complex tissue structures in H&E images introduce substantial artifacts, obscuring crucial pathological features within background noise and hindering precise feature extraction; (ii) Current approaches inadequately model the non-linear correlations inherent in spatially adjacent gene expression, underutilizing spatial dependency information; (iii) They struggle to effectively mitigate noise interference and the inherent weak correlations between histological images, gene expression, and spatial positions. Collectively, these factors constrain the accuracy of spatial gene expression prediction.

To address the aforementioned challenges, this study proposed DFCE-KanT, a novel contrastive learning-based model for spatial gene expression prediction. Specifically: (i) In the image feature extraction branch, we designed a Feature Channel Enhancement (FCE) attention mechanism to enhance critical pathological features while suppressing noise; (ii) For gene feature processing, we constructed a KanT module to adaptively capture the non-linear correlations of gene expression across spatial locations; (iii) In the cross-modal projection stage, we introduced a Kolmogorov-Arnold Network (KAN), which dynamically corrects the heterogeneous feature distributions between H&E images and spatial gene expression through learnable B-spline basis functions. We evaluated DFCE-KanT on five ST datasets: breast cancer HER2+ cutaneous squamous cell carcinoma (cSCC), breast cancer Alex, HBC, and Liver dataset. Experimental results demonstrate that DFCE-KanT outperforms existing methods, achieving superior performance in predicting spatial gene expression from histological images.

## 2 Materials and methods

### 2.1 Dataset and pre-processing

We evaluated DFCE-KanT on five publicly available multi-slice ST datasets covering both tumor and normal tissues. All datasets comprised spatially resolved gene expression data, corresponding spatial coordinate, and histology images. Specifically, The HER2+ dataset (Andersson *et al.* 2021) comprises 32 tissue slides from seven patients, each with at least 180 data points. The cSCC dataset (Ji *et al.* 2020) consists of 12 tissue sections from four patients, with three slides per patient. The Alex dataset (Wu *et al.* 2021) and the 10× Genomics (Janesick *et al.* 2023) HBC dataset include six and three breast cancer tissue samples. The Liver dataset (Andrews *et al.* 2024) consists of four normal human liver samples.

The ST dataset was preprocessed as follows. In case of histology image, given the huge size of WSIs and the high computational cost, image segmentation was used (Aresta *et al.* 2019, Dimitriou *et al.* 2019, Chen *et al.* 2022). We took a patch with dimension of 224 × 224 pixels from an H&E stained slide around the spatial coordinate of each Visium spot. This patch size approximates the spot diameter, ensuring precise alignment between local histological features and corresponding gene expression profiles. Regarding gene expression data, we filtered and normalized the data by selecting top 1000 highly variable genes (HVGs) per section, in order to mitigate the noise. We removed genes which are expressed on fewer than 1000 spots over all training sections. Counts for each spot were normalized by the total counts of the spot, scaled by 10^6^, and log-transformed by log⁡(x+1) to stabilize variance and lessen distribution skewness.

### 2.2 Overview of DFCE-KanT

**Figure 1:**
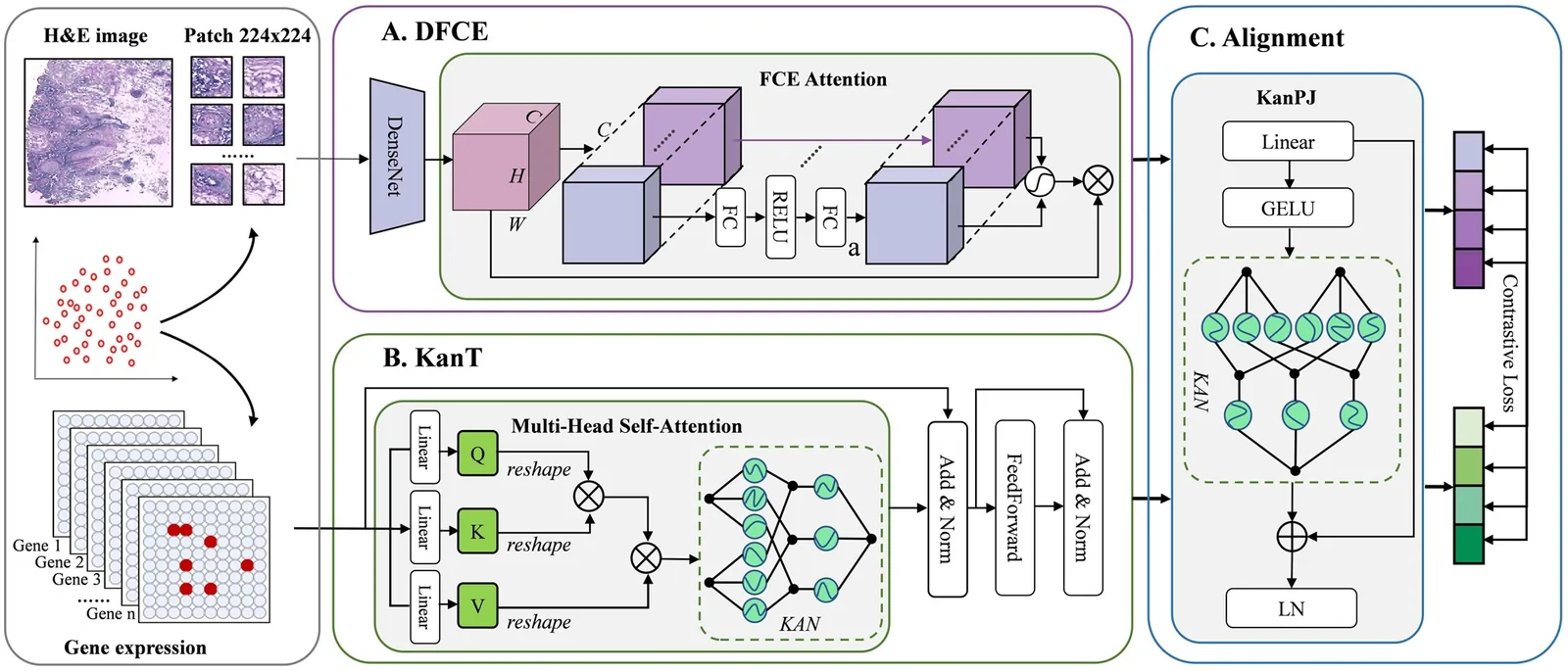
Architecture of DFCE-KanT. There are three parts in total: DFCE, KanT, and Alignment.

We presented DFCE-KanT, a new deep learning architecture for the problem of spatially resolved gene expression prediction directly from histology images. In order to exploit all available complimentary information over the different modalities, the model learned non-linear dependencies between tissue morphologies, gene expression patterns, and spatial location information. As shown in Fig. 1, our DFCE-KanT consists of three major components: (i) an image feature extractor DFCE module,(ii) a KanT module to integrate the gene expression with the spatial features extraction, and (iii) a feature alignment module to bridge heterogenous modalities.

#### 2.2.1 The image feature extraction module (DFCE)

This module captured essential histomorphological features from H&E-stained image patches, which were cropped around each sequencing spot and used as input. Inspired by the attention (Vaswani *et al.* 2017), the DFCE module adopted DenseNet-121 as its backbone network and incorporates a Feature Channel Enhancement (FCE) attention mechanism. The FCE mechanism adaptively re-weighted feature channels to amplify the most informative morphological patterns while suppressing irrelevant noise. Its primary objective was to improve the quality of the learned representations by explicitly modeling interdependencies among convolutional feature channels.

Although pathological images contained distinctive cellular and tissue level details, whole slide images often included substantial redundant information. While DenseNet preserved fine details through dense feature reuse, it lacked the capacity to prioritize the most discriminative features. To mitigate this limitation, we integrated the FCE attention mechanism with DenseNet-121 for pathological image feature extraction. This hybrid design enhanced the representational capacity of pathological features via channel-wise attention.

The procedure is as follows: DenseNet-121 generated a multi-channel feature map X from each input image patch. Since the importance of individual feature channels varied, X was subsequently refined by the FCE attention mechanism. As illustrated in Fig. 1, the FCE mechanism first modeled nonlinear channel relationships through a fully connected (FC) layer. A ReLU activation function was applied to enhance feature sparsity, followed by a second FC layer that estimated channel importance. Finally, a Sigmoid activation function generated channel attention weights A. These weights were multiplied element-wise with the original features, thereby emphasizing salient patterns and attenuating less relevant ones.


(1) A=σ(FC(δ(FC(X))))

(2) $$E_{x}=A⊗X$$

where X denotes the feature map from DenseNet-121, FC represents a fully connected layer. δ is the ReLU nonlinear activation function, σ is the Sigmoid activation function, and ⊗ indicates element-wise multiplication. The output $E_{x}$ corresponds to the enhanced feature representation for each image patch.

#### 2.2.2 Gene expression and spatial feature extraction module (KanT)

Spatial gene expression data possess the properties of a large number of dimensions with heavy noise. In order to deal with the problem of sparseness as well as outliers, We integrated Kolmogorov-Arnold Networks (Kan) (Liu *et al.* 2025) into the Transformer architecture, constructing a spatially aware feature encoder named KanT. This encoder adaptively learned interactions among genes and their spatial dependencies, effectively capturing the spatial heterogeneity of gene expression. The gene expression profile $g_{i}\in R^{d}$ and spatial coordinates $p_{i}=(x_{i},y_{i})$ of each spot were received by the module as joint input.

First, the gene features and coordinate embeddings were concatenated to form a fused representation:


(3) $$Z={\left[g_{1}⊕p_{1},g_{2}⊕p_{2},\ldots ,g_{n}⊕p_{n}\right]}^{T}$$

Where $Z\in R^{n\times d}$ is the fused feature, n is the number of spots and d the feature dimension.

A multi-head attention (MHA) mechanism then models interactions among spots. For head i:


(4) $${\mathrm{head}}_{i}=\mathrm{Attention}(ZW_{i}^{Q},ZW_{i}^{K},ZW_{i}^{V})$$

(5) $$\mathrm{Attention}(Q,K,V)=\text{softmax}(\frac{QK^{T}}{\sqrt{d}})V$$

Here, $W_{i}^{Q}$, $W_{i}^{K}$, $W_{i}^{V}$ are projection matrices, and d is the head dimension. Next, the outputs of all h heads were concatenated and passed through a KAN layer. Based on KAN, some techniques have made several of advancements and made notable advances (Drokin 2024, Firsov *et al.* 2024). The KAN layer employed learnable spline basis functions to capture nonlinear gene interactions:


(6) $$\mathrm{MHS}(Z)=\mathrm{KAN}(\mathrm{Concat}({\mathrm{head}}_{1},{\mathrm{head}}_{2},\ldots ,{\mathrm{head}}_{h}))$$

Finally, a feedforward network (Bebis and Georgiopoulos 1994) performed nonlinear transformation and dimensionality reconstruction, producing the integrated feature vector $E_{z}$ that encodes both gene expression and spatial information.

#### 2.2.3 The feature alignment module

The morphological features in histological images and spatial gene expression exhibit multiscale nonlinear relationships. Conventional linear models often failed to capture the co-regulatory mechanisms between genes and morphology within the microenvironment, especially for genes with gradient expression patterns. To address this, motivated by previous approaches (Al-Qaness and Ni 2025, Li *et al.* 2025), we designed the KanPJ projection head to map $E_{x}$ (the image features extracted from DFCE) and $E_{z}$ (the gene features encoded by KanT) into a unified latent space. First, they were linearly projected to align dimensions, yielding $E_{x}^{\prime }$ and $E_{z}^{\prime }$ as the basis for spatial alignment. Subsequently, a nonlinear activation function was introduced to adapt to the zero-inflated distribution of gene expression and to fit the complex relationship between image features and genes. Spatial expression patterns were further captured using the B-spline basis functions in the KAN. Residual connections mitigated deep network degradation and prevented the loss of key biological signals.

Finally, layer normalization was applied to standardy feature distributions and eliminate batch effects, generating aligned features $F_{x}$ and $F_{z}$ for subsequent contrastive learning (Chen *et al.* 2020, Le-Khac *et al.* 2020). The KanPJ projection head is formulated as follows:


(7) $$F_{x}=LN(\mathrm{KAN}(\mu (E_{x}^{\prime }))+E_{x}^{\prime })$$

(8) $$F_{z}=LN(\mathrm{KAN}(\mu (E_{z}^{\prime }))+E_{z}^{\prime })$$

where μ denotes the Gaussian Error Linear Unit (GELU) activation function, KAN represents the Kolmogorov-Arnold network, and LN indicates layer normalization.

This module was then trained end-to-end within a contrastive learning framework to establish similarity relationships between images and genes. Through cross-modal representation alignment, the model learned latent associations between morphological features and gene modules. Specifically, in the embedding space, the cosine similarity between image features and gene expression features from the same sequencing spot was maximized, while the similarity between embeddings from different spots was minimized.


(9) $$S=\frac{\mathrm{sim}(F_{x},F_{z})}{\tau }$$

(10) $$\mathrm{Los}s_{x\to z}=-\frac{1}{N}\sum_{i=1}^{N}\log\frac{\;{\text{exp}}^{S_{ii}}}{\sum_{j=1}^{N}\;{\text{exp}}^{S_{ij}}}$$

(11) $$\mathrm{Los}s_{z\to x}=-\frac{1}{N}\sum_{i=1}^{N}\log\frac{\;{\text{exp}}^{S_{ii}}}{\sum_{j=1}^{N}\;{\text{exp}}^{S_{ji}}}$$

(12) $$\mathrm{Los}s_{\mathrm{total}}=\frac{1}{2}(\mathrm{Los}s_{x\to z}+\mathrm{Los}s_{z\to x})$$

Here, the similarity matrix S is derived from cosine similarity (sim), τ is a temperature coefficient, and N is the training batch size. $\mathrm{Los}s_{x\to z}$ measures the similarity between each image embedding and its corresponding genes, ensuring that tissue structure aligns with its specific gene expression profile. $\mathrm{Los}s_{z\to x}$ measures the similarity between each gene embedding and its matching images, emphasizing that gene expression should be associated with the morphological features of its native spatial context. $\mathrm{Los}s_{\mathrm{total}}$ denotes the overall contrastive loss.

This contrastive loss (Radford *et al.* 2021) spatially aligned tissue regions with similar morphology to those with analogous gene expression patterns. By framing the task as implicit association modeling, DFCE-KanT learned a unified aligned embedding space. Once trained, the model predicts spatial gene expression from H&E image tiles via a KNN-based retrieval strategy. The optimal K is set based on sensitivity analysis (Fig. S1, available as supplementary data at *Bioinformatics* online). For a given test image tile, it retrieves known expression profiles that are similar in the learned embedding space to infer the corresponding gene expression.

### 2.3 Evaluation metrics

We used Pearson Correlation Coefficient (PCC), Mean Squared Error (MSE), Mean Absolute Error (MAE), and Structural Similarity Index Measure (SSIM) to compare the performance of DFCE-KanT and other baseline methods. These metrics were calculated using the following equation.


(13) $$\mathrm{PCC}=\frac{E[\left(x_{i}-\mu_{i})({\hat{x}}_{i}-{\hat{\mu }}_{i}\right)]}{\sigma_{i}{\hat{\sigma }}_{i}}$$

(14) $$\mathrm{MSE}=\frac{1}{N}\sum_{i=1}^{N}{\left(x_{i}-{\hat{x}}_{i}\right)}^{2}$$

(15) $$\mathrm{MAE}=\frac{1}{N}\sum_{i=1}^{N}\left|x_{i}-{\hat{x}}_{i}\right|$$

where $x_{i}$ and ${\hat{x}}_{i}$ denote the spatial expression vectors of gene i in the ground truth and the predicted result, respectively. $\mu_{i}$ and ${\hat{\mu }}_{i}$ represent the average expression values of gene i in the ground truth and the predicted result, respectively. $\sigma_{i}$ and ${\hat{\sigma }}_{i}$ denote the standard deviations of the spatial expression of gene i in the ground truth and the predicted result, respectively.


(16) $$\mathrm{SSIM}=\frac{\left(2\mu_{x}\mu_{y}+{\left(k_{1}L\right)}^{2})(2\sigma_{xy}+{\left(k_{2}L\right)}^{2}\right)}{\left(\mu_{x}^{2}+\mu_{y}^{2}+{\left(k_{1}L\right)}^{2})(\sigma_{x}^{2}+\sigma_{y}^{2}+{\left(k_{2}L\right)}^{2}\right)}$$

where $\mu_{x}$ and $\mu_{y}$ are the mean intensities of images x and y, $\sigma_{x}^{2}$ and $\sigma_{y}^{2}$ are the variances, $\sigma_{xy}$ is the covariance. The constants $k_{1}$ and $k_{2}$ are 0.01 and 0.03, respectively, to stabilize the division. L is the dynamic range of pixel values.

PCC measured the average correlation for each gene type, considering ground truth and predictions for every slide image. For each tissue section, we calculated the PCC for all highly variable genes (HVG) and the top 50 highly expressed genes (HEG). Meanwhile, for each gene type in each slide image, MSE and MAE measured the sample deviation between predictions and ground truth. SSIM is a perceptual metric that quantifies data quality degradation caused by processing such as data compression or transmission. SSIM helped evaluate the model’s ability to preserve the spatial patterns and structural features of gene expression (Wang *et al.* 2004). For PCC and SSIM, higher values indicated better performance. Conversely, for MSE and MAE, lower values indicate better performance.

## 3 Results

### 3.1 Experiment settings

For DFCE-KanT, we adopted the Adam optimizer with the learning rate of 4 × 10^−4^, weight decay to 1 × 10⁻³, and batch size to 512. Training was conducted for 90 epochs on the HER2+ and cSCC datasets, 60 epochs on the Alex and HBC datasets, and 30 epochs on the Liver dataset (Fig. S2, available as supplementary data at *Bioinformatics* online). To ensure a fair comparison, all experiments for the baseline methods (THItoGene, HisToGene, His2ST, BLEEP, mclSTExp) and our DFCE-KanT were performed on same NVIDIA Tesla V100 GPUs.

To evaluate model performance, we performed leave-one-out cross-validation on the five ST datasets (HER2+, cSCC, Alex, HBC and Liver). In each fold, one tissue section was kept out for testing, and the remaining sections were used for training. This process was repeated until every section had served as the test set once.

### 3.2 Performance comparison with baseline methods

**Figure 2:**
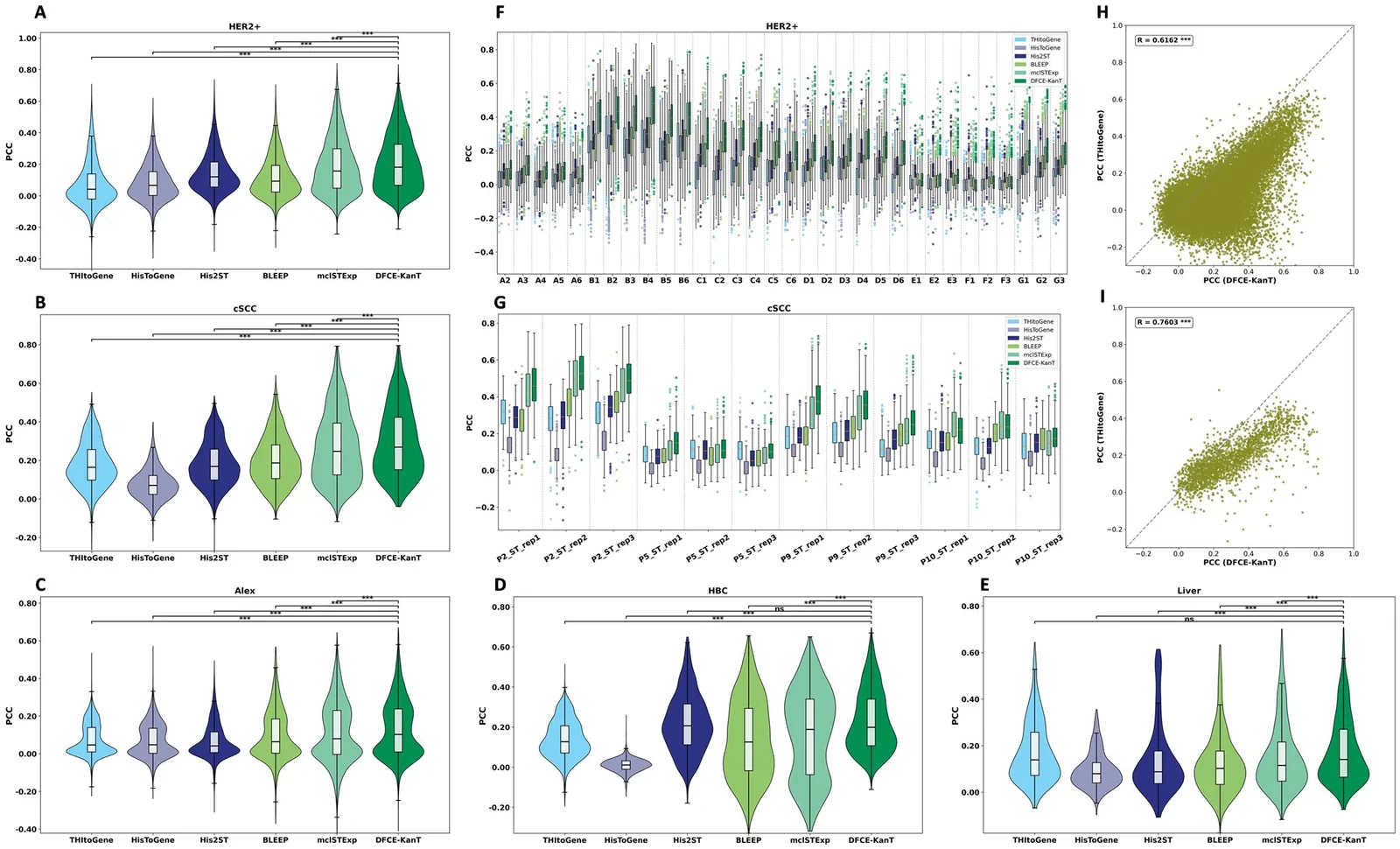
Comparative evaluation of spatial transcriptome prediction methods. Violin plots of PCC distributions between observed and predicted gene expression across the HER2+, cSCC, Alex, HBC, and Liver datasets for all six methods (THItoGene, HisToGene, His2ST, BLEEP, mclSTExp, and DFCE-KanT). PCC comparisons of gene expression prediction results on the (A) HER2+, (B) cSCC, (C) Alex, (D) HBC, and (E) Liver datasets. Section-level PCC value on the (F) HER2+ and (G) cSCC datasets.

By calculating the Pearson correlation coefficient (PCC) between predicted and observed expression in each tissue section, DFCE-KanT was evaluated against five leading methods for spatial gene expression prediction. This metric quantitatively evaluates the accuracy of spatial expression pattern reconstruction. As shown in Fig. 2A–E, DFCE-KanT achieves higher PCC values than all baseline methods across almost all five datasets. In the HER2+ dataset, DFCE-KanT (PCC = 0.2071) outperforms THItoGene (0.0715, +189.65%), HisToGene (0.0832, +148.92%), Hist2ST (0.1443, +43.52%), BLEEP (0.1182, +75.21%), and mclSTExp (0.1863, +11.16%). Consistent improvements are observed across the remaining datasets. cSCC (+65.47%, +285.68%, +62.75%, +48.55%, +9.38%), Alex (+79.18%, +90.12%, +97.26%, +34.80%, +17.84%), HBC (+65.04%, +2340.43%, +4.32%, +64.01%, +40.74%), and Liver (+5.13%, +92.23%, +31.18%, +46.78%, +20.17%). Wilcoxon signed-rank test results further verify that DFCE-KanT yields significant performance improvements over most baseline methods (P<.001).

We further assessed section-level stability by visualizing PCC per tissue section. DFCE-KanT achieved the highest PCC in 19/32 sections of the HER2+ dataset (Fig. 2F, Table S1, available as supplementary data at *Bioinformatics* online) and 10/12 sections of the cSCC dataset (Fig. 2G, Table S2, available as supplementary data at *Bioinformatics* online). Section-wise results for Alex, HBC and Liver datasets are provided in Supplementary Materials (Figs. S3–S5, Tables S3–S5). These findings confirm its stable predictive performance across most tissue sections.

### 3.3 Visualization of gene expression

To characterize which genes DFCE-KanT predicts most accurately, we calculated the PCC and associated *P*-value between predicted and observed expression for each gene. Genes were ranked by their average − log10(*P*-value) across all sections, and the top 10 genes with the lowest average *P*-values were selected for further analysis.

**Figure 3:**
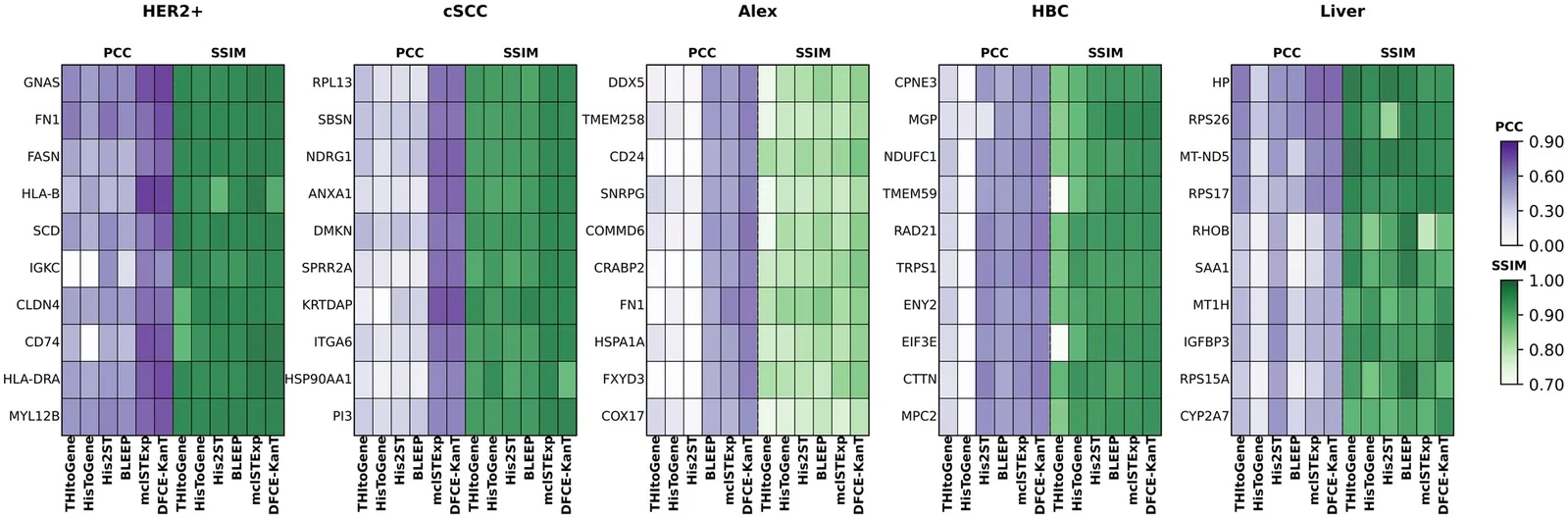
PCC and SSIM evaluation of the top 10 predicted genes across HER2+, cSCC, Alex, HBC and Liver datasets, comparing DFCE-KanT with baseline methods including THItoGene, HisToGene, His2ST, BLEEP and mclSTExp.

PCC and SSIM heatmaps for the top 10 genes predicted by all six methods are shown in Fig. S7, available as supplementary data at *Bioinformatics* online. For all datasets, DFCE-KanT achieved the best PCC and SSIM results. We further evaluated other methods using the top 10 genes identified by DFCE-KanT as a unified reference. As illustrated in Fig. 3, DFCE-KanT achieved the highest PCC and SSIM values in all tests, demonstrating its robustness in capturing biologically relevant genes. Most top-ranked genes predicted by DFCE-KanT also align well with those reported in previous studies (Pang *et al.* 2021, Zeng *et al.* 2022, Jia *et al.* 2024, Min *et al.* 2024), indicating strong biological relevance. Collectively, these results verify that our model can reliably identify disease-associated gene expression patterns.

**Figure 4:**
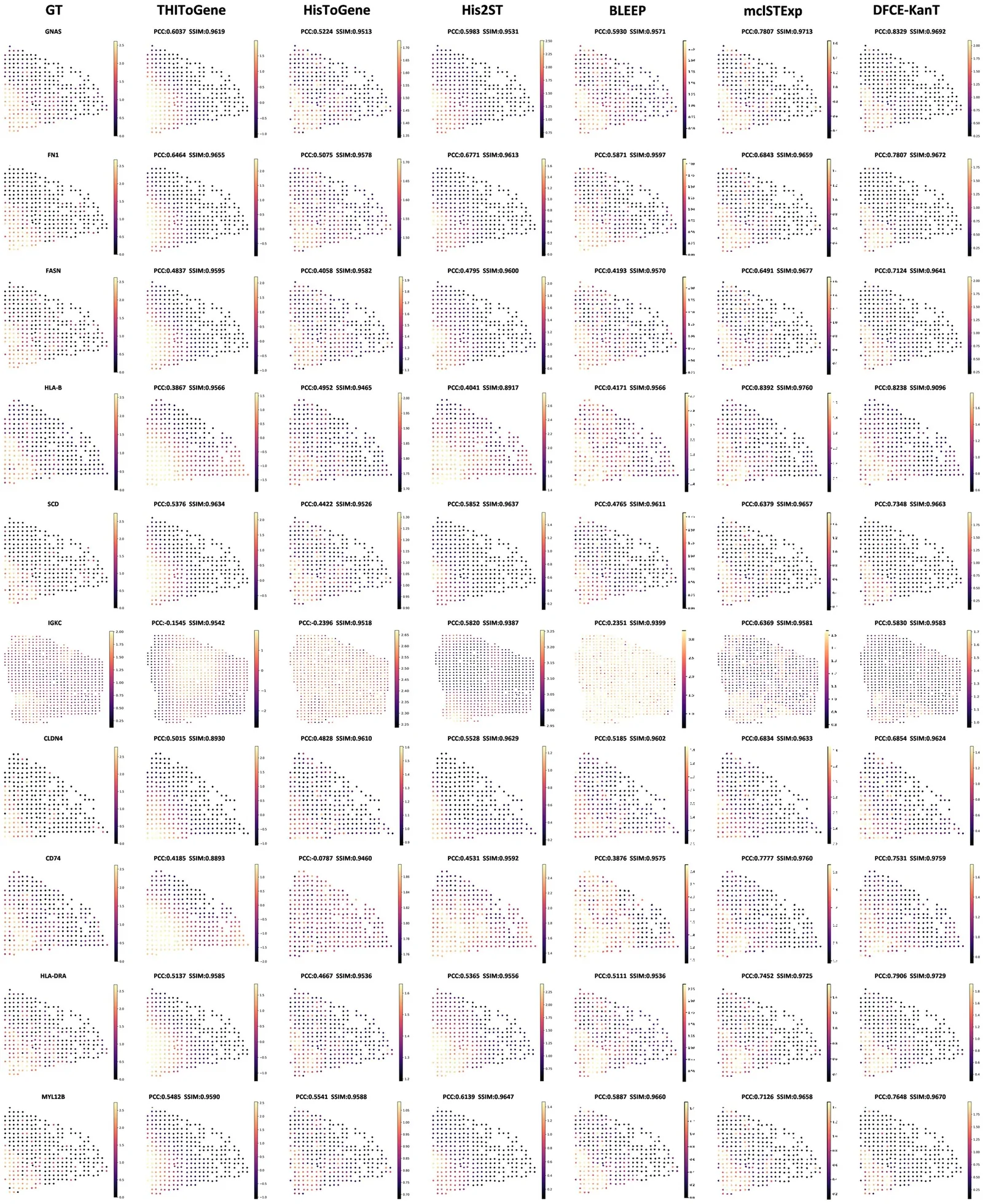
The top 10 predicted genes with the highest mean − log10(*P*-value) on the HER2+ dataset. The ground-truth gene expression is shown in the first column, and gene expression predictions from six different methods are shown in the final six columns.

For a clear visual comparison, Fig.  4 displays the predicted and true expression patterns of the top 10 genes in the HER2+ dataset. Our DFCE-KanT predictions most closely match the real spatial patterns. DFCE-KanT continuously outperforms the baseline methods. Parallel studies in cSCC, Alex, HBC and Liver datasets were yielded consistent results (Tables S6–S9, available as supplementary data at Bioinformatics online).

To assess how well each method preserves spatial tissue architecture, we applied K-means clustering to predicted expression from all methods on expert-annotated HER2+ sections (B1, C1, D1, E1, F1, G2). SSIM was used to measure the extent of similarity between the clustering results and the true pathological structure.

**Figure 5:**
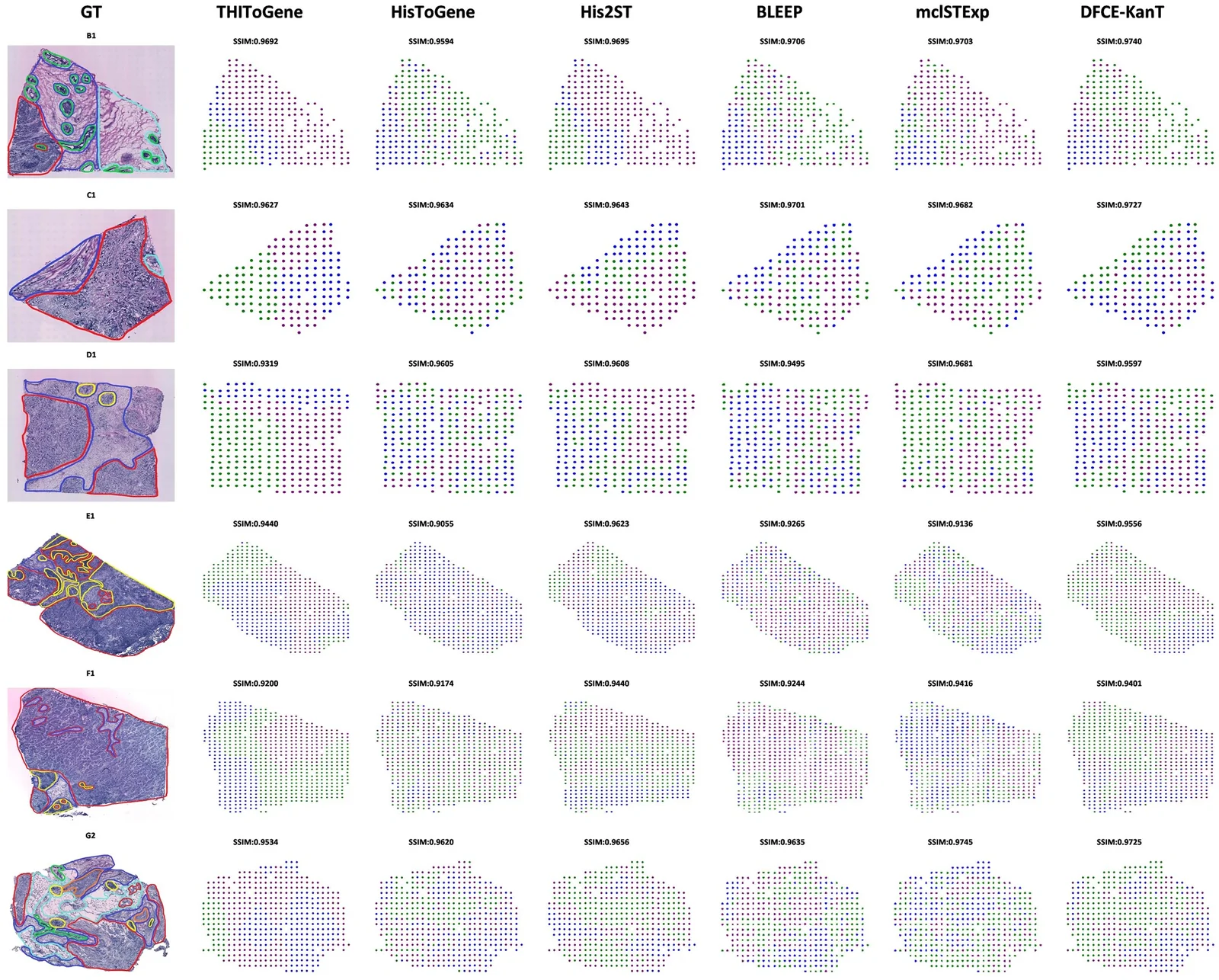
Comparison of spatial structure preservation in HER2+ tissue sections via K-means clustering. Spatial domain assignments predicted by THItoGene, HisToGene, His2ST, BLEEP, mclSTExp and DFCE-KanT are overlaid on H&E-stained images, with SSIM applied to quantify similarity to expert annotations. The first column presents histology images labeled by pathologists, and the remaining six columns show clustering results of gene expression from each method.

As shown in Fig.  5, the cluster assignments are overlaid as color-coded maps on the corresponding H&E images. DFCE-KanT achieves the highest SSIM on B1 and C1. On B1, it outperforms THItoGene, HisToGene, His2ST, BLEEP and mclSTExp by 0.49%, 1.52%, 0.46%, 0.35% and 0.38%, respectively. On C1, it surpasses these methods by 1.04%, 0.97%, 0.88%, 0.27% and 0.47%, respectively. On G2, mclSTExp obtains the best result, while DFCE-KanT outperforms all other baselines. On D1, E1 and F1, DFCE-KanT delivers competitive performance and consistently outperforms THItoGene and HisToGene. Overall, our model reconstructs spatial gene expression with high fidelity and effectively captures tissue spatial features.

### 3.4 Ablation experiment

To verify the contribution of each component in DFCE-KanT, we carried out systematic ablation experiments on its three main modules: the FCE attention mechanismin in DFCE module, the KanT correlation module, and the Alignment projector. Experiments were performed on the HER2+, cSCC, Alex, HBC, and Liver datasets. The Pearson correlation coefficient (PCC) for the top 50 highly expressed genes (HEG) and all highly variable genes (HVG), together with the mean squared error (MSE) and mean absolute error (MAE) over all predicted spots, were used to assess the prediction accuracy.

**Figure 6:**
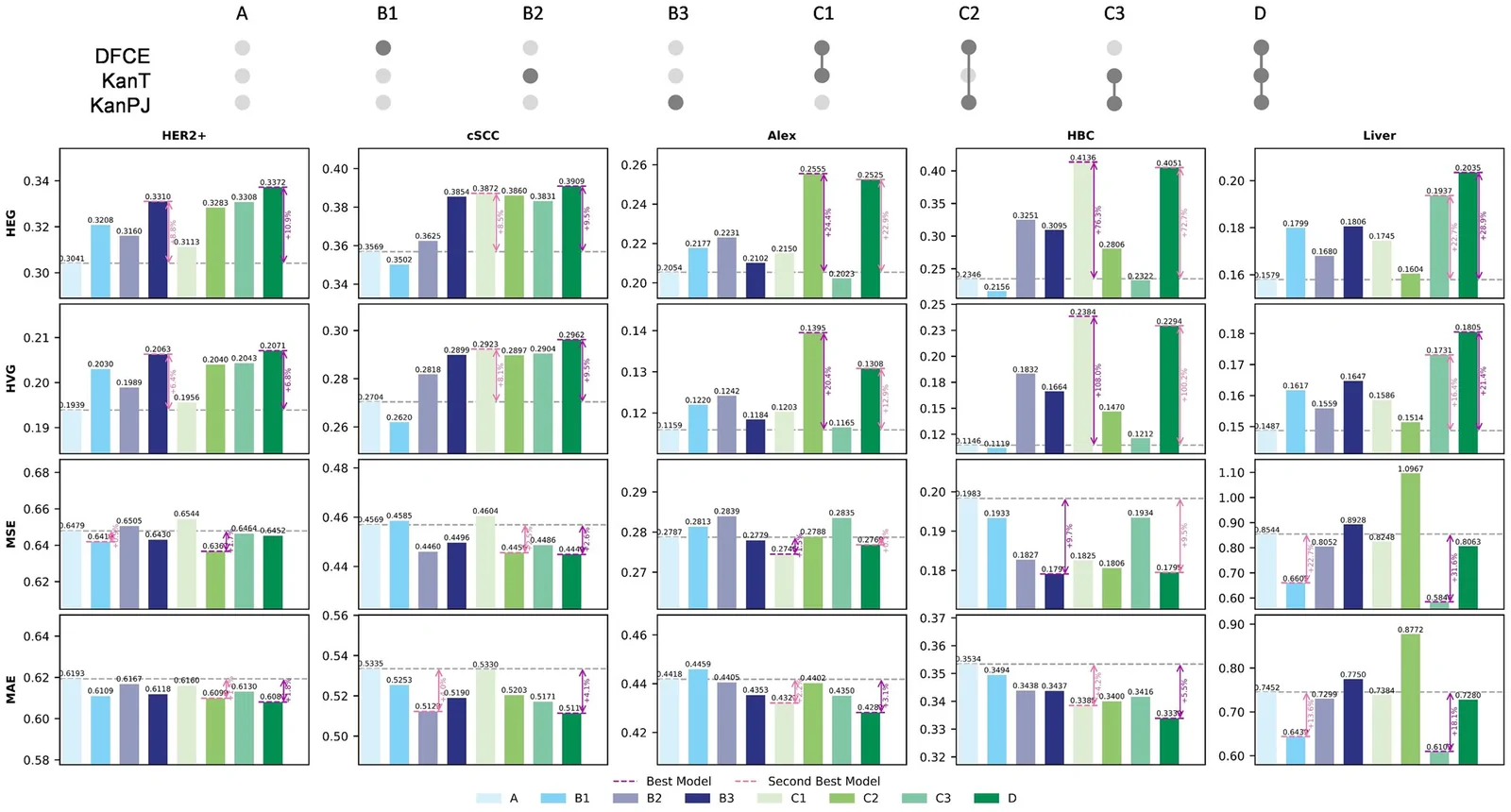
Ablation study of latent embedding module.

As shown in Fig.  6. Ours DFCE-KanT (Model D) achieved best in 11/20 metrics (3 for HER2+, 4 for cSCC, 1 each for Alex and HBC, and 2 for Liver) and second-best in 6/20 metrics (3 each for Alex and HBC). Performance deteriorated when any module was removed, as seen by lower HEG and HVG together alongside higher MSE and MAE. Detailed observations are as follows.

- Removing the FCE attention mechanism in DFCE module (Model C3 versus Model D) noticeably decreased HEG and HVG while raising MSE and MAE on all datasets except Liver. Comparing Model B2 with C1 and Model B3 with C2 revealed a similar trend.
- Removing the Kan network in KanT (Model C2 versus Model D) lowered HEG and HVG, while raised MSE and MAE on all datasets except Alex. Comparing Model B1 with C1 and Model B3 with C3 showed comparable declines.
- Removing the Alignment module (Model C1 versus Model D) significantly reduced HEG and HVG and increased MSE and MAE on datasets other than cSCC and HBC. This pattern was also evident between Model B1 and C2 and between Model B2 and C3.
- Overall module contribution was assessed using a baseline model (Model A) that omitted the crucial aspect of each module. Relative to Model A, our Model D improved HEG by 10.9%, 9.5%, 22.9%, 72.7% and 28.9% for HER2+, cSCC, Alex, HBC and Liver datasets, and HVG by 6.8%, 9.5%, 12.9%, 100.2% and 21.4%. For the four datasets excluding Liver, MSE dropped by 0.4%, 2.6%, 0.7% and 9.5%, while MAE declined by 1.8%, 4.1%, 3.1% and 5.5%.

The results indicate that each of the DFCE-KanT modules solves one particular space gene-expression prediction problem, and the DFCE module can extract information at a finer granularity from HE images; the KanT module can efficiently capture the spatial heterogeneity of gene expression, while the Alignment module ensure an effective multimodal features alignment. Together, these aspects boost the accuracy of spatial gene expression prediction from histology images.

In a nutshell, DFCE-KanT achieved the best or second best results in almost all five dataset which proved that it was excellent and robust. Efficacy of our proposed architecture is also justified through ablation studies, that show how the three designed modules complement each other and tackle the main challenges of spatial gene expression prediction from histology images.

## 4 Discussion

Predicting spatial gene expression from histology images depends on strong correlations between image features and gene expression data. We develop DFCE-KanT to solve key challenges in this cross-modal prediction task.

The DFCE module adopts an FCE attention mechanism. It adjusts weights across feature channels to highlight valuable tissue morphology and filter out noise. This design extracts pathological features more precisely than classic attention methods including SE (Hu *et al.* 2020) and CBAM (Woo *et al*. 2018) (Table S10, available as supplementary data at *Bioinformatics* online). The KanT encoder integrates Kolmogorov-Arnold Networks (KAN) and Transformer. It efficiently captures spatial heterogeneity in gene expression. Traditional MLPs and standard Transformer cannot well model complex nonlinear relationships. Model performance drops noticeably when we replace KAN with these conventional components across all datasets (Table S10, available as supplementary data at *Bioinformatics* online). We build the KanPJ projection head based on KAN. It projects image and gene expression features into a unified embedding space. This approach outperforms linear alignment and retains vital biological information. Control experiments (Table S11, available as supplementary data at *Bioinformatics* online) prove that our contrastive learning strategy is superior to direct regression. It also validates the effectiveness of our cross-modal alignment design.

The three core modules work collectively to minimize cross-modal biases and yield robust joint features. We assessed DFCE-KanT across five public datasets: HER2+, cSCC, Alex, HBC and Liver. The model ranked first or second on most metrics, demonstrating solid predictive power and good generalization across datasets. A series of ablation tests validated the contribution of each component. Eliminating any module lowered PCC values for highly expressed genes (HEG) and highly variable genes (HVG), and increased MSE and MAE. Against the baseline model, DFCE-KanT lifted HEG by 10.9%–72.7% and HVG by 6.8%–100.2%. It also reduced MSE by up to 9.5% and MAE by up to 5.5%. Every module fulfills a dedicated role. The FCE mechanism refines histopathological feature extraction. The KanT encoder identifies spatial patterns in gene expression. The alignment module fuses multimodal data efficiently. Their joint operation markedly improves overall prediction performance.

Spatial transcriptomic experiments are costly and time-consuming. These limitations hinder their large-scale clinical application. Our method enables fast and low-cost prediction using routine histopathological slides. It helps researchers understand disease mechanisms and assists pathologists in diagnosis, staging and prognosis. It also provides a solid foundation for personalized clinical treatment.

Although DFCE-KanT has a promising prospect in predicting spatial gene expression, several limitations remain to be addressed. We outline three directions for future improvements. First, public spatial transcriptomic datasets contain relatively few patients, which prevents large-scale model training. This study adopted leave-one-out cross-validation at the tissue section level. This setting follows common practices in related studies and ensures fair comparison with published baselines. To boost model generalization, we will collect and integrate available spatial transcriptomic data, expand our dataset with larger patient cohorts and perform further validation. Second, as single-cell resolution spatial transcriptomic data becomes more available, we will build models to support gene expression prediction at finer spatial and cellular resolutions. Third, we will incorporate multi-omics data and broaden clinical applicability of the model.

## Supplementary Material

btag502_Supplementary_Data

## Data Availability

This study utilized five publicly available ST datasets. The human HER2-positive breast tumor ST data can be found at https://github.com/almaan/her2st/. The Human cutaneous squamous cell carcinoma data are available from GSE144240. The Alex data and HBC data can be downloaded from https://doi.org/10.48610/4fb74a9. The Liver data are available from GSE240429.
