# Supplementary material for: DFCE-KanT: predicting spatial gene expression from histology images via contrastive learning
Source: Bioinformatics. 2026 Jul 8;42(8):btag502. doi: 10.1093/bioinformatics/btag502 (PMC13499228; doi:10.1093/bioinformatics/btag502)
Supplement: Supplementary file 1 [file btag502_supplementary_data.pdf]

Supplementary Materials

DFCE-KanT: Predicting Spatial Gene Expression from Histology Images via Contrastive Learning

Fang Li[1,‡], Pengyu Wang[1,‡], Junjie Shen[2,1], Yazhou Wu[1,*]

[1] Department of Health Statistics, College of Preventive Medicine, Army Medical University, Chongqing 400038, China.

[2] Center for Medical Big Data and Artificial Intelligence, Southwest Hospital, the First Affiliated Hospital of the Army Military Medical University, Chongqing, 400038, China.

[*]Corresponding author. Department of Health Statistics, College of Preventive Medicine, Army Medical University, No. 30, Gaotanyan Zhengjie, Shapingba District, Chongqing 400038, China. E-mail: asiawu@tmmu.edu.cn

[‡]Fang Li and Pengyu Wang contributed equally to this work

1 Supplementary Notes

1.1 Supplementary Note:Sensitivity Analysis

The DFCE-KanT model uses a KNN retrieval strategy to infer gene expression for test samples. Specifically, it retrieves known expression profiles from the learned embedding space. We performed a sensitivity analysis for the KNN parameter K across five datasets (HER2+, cSCC, Alex, HBC and Liver) to clarify inference details and assess parameter effects. Model performance was quantified via four metrics: HEG, HVG, MSE and MAE. All results are presented in **Fig. S1**. We selected the optimal K for each dataset based on comprehensive trade-offs between correlation, prediction accuracy and model stability.

For HER2+, MSE and MAE bottom out at K = 200 and rise as K increases. HEG and HVG rise sharply before K = 200 and then grow moderately. We thus set K = 200 for this dataset.

For cSCC, PCC gradually drops when K> 300, MSE rises for K > 400, and MAE worsens consistently with larger K. At K = 600, correlation remains strong and prediction errors show no notable increase. This value delivers balanced performance across all metrics, so we adopted K = 600.

For Alex, correlation improves steadily with increasing K, while prediction errors begin to rise after K reaches 800. At K = 2400, HEG and HVG stay high, accompanied by only minor growth in MSE and MAE. This setting achieves a favorable balance of correlation, precision and stability, and was used in all subsequent experiments.

For HBC, HEG and HVG peak at K = 200 and decline slightly afterwards. MAE plateaus beyond this point, and the reduction of MSE slows markedly. We chose K = 200 by jointly considering correlation, prediction accuracy and computational efficiency.

For Liver, PCC decreases and error metrics keep falling as K grows, revealing a clear trade-off between correlation and prediction error. At K = 200, the model retains good correlation ability and achieves much lower prediction errors compared with smaller K values. A smaller K improves correlation but degrades precision, whereas a larger K further reduces errors marginally at the cost of sustained correlation loss. We finally adopted K = 200 to reconcile these two aspects.

1.2 Supplementary Note: Convergence Analysis

We defined dataset-specific training epochs according to model convergence. Variations in data size, complexity and distribution lead to different convergence trends across HER2+, cSCC, Alex, HBC and Liver datasets. We used 90 epochs for HER2+ and cSCC, 60 epochs for Alex and HBC, and 30 epochs for Liver. **Fig. S2** shows the corresponding loss curves and verifies full convergence for all datasets.

1.3 Supplementary Note: Ablation Study on the Core Components of DFCE-KanT

To quantitatively evaluate the contributions of the proposed KanT module and FCE attention module, we conducted comprehensive ablation experiments by replacing each component with mainstream alternatives, while keeping the rest of the architecture unchanged. The results across five datasets (HER2+, cSCC, Alex, HBC, Liver) are presented in **Table S10**.

a. Effectiveness of the KanT Module

We compared the performance when replacing the embedded KAN block with two conventional feed-forward designs: MLP and Transformer feed-forward.

Correlation performance (HEG and HVG, ↑): The full DFCE-KanT model consistently outperforms both MLP and Transformer feed-forward across all datasets, achieving the highest PCC values for both highly expressed genes (HEG) and highly variable genes (HVG). The most substantial improvements are observed on the HBC dataset, where DFCE-KanT yields 31% higher HEG (0.4051 vs. 0.3080) and 32% higher HVG (0.2294 vs. 0.1740) compared to the best baseline (+MLP). This indicates that the KAN-based design is more effective at capturing the complex nonlinear relationships among spatially resolved gene expression profiles.

Prediction error (MSE and MAE, ↓): DFCE-KanT achieves the lowest MSE and MAE on three out of five datasets (cSCC, Alex, HBC). On the remaining two datasets (HER2+, Liver), its error metrics remain comparable to the best baseline while maintaining significantly higher correlation. This confirms that the KanT module improves predictive consistency without sacrificing prediction accuracy.

b. Effectiveness of the FCE Attention Module

We benchmarked our proposed FCE attention against two widely used channel attention mechanisms: SE and CBAM.

Correlation performance (HEG and HVG, ↑): DFCE-KanT with FCE attention outperforms both SE and CBAM variants on all datasets. For example, on the cSCC dataset, FCE improves HEG by 11.6% (0.3909 vs. 0.3503) and HVG by 11.6% (0.2962 vs. 0.2655) over the best baseline (CBAM). This suggests that FCE attention better identifies and focuses on informative features relevant to gene expression prediction.

Prediction error (MSE and MAE, ↓): The full model obtains the lowest MSE and MAE on cSCC, Alex, and HBC datasets, outperforming both SE and CBAM. Although CBAM achieves slightly lower errors on the Liver dataset, its correlation performance is substantially lower than DFCE-KanT. Overall, FCE attention leads to a better balance between correlation and prediction accuracy.

Ablation studies confirm KanT and FCE attention jointly boost DFCE-KanT performance.

1.4 Supplementary Note: Ablation Study on the Core Designs of DFCE-Kan

We performed two sets of targeted control experiments, as summarized in **Table S11**: (1) replacing the proposed KAN module with a standard MLP baseline; and (2) comparing our contrastive learning objective with a direct regression objective. All experiments are conducted under identical settings across five spatial transcriptomics datasets (HER2+, cSCC, Alex, HBC, Liver).

a. Effectiveness of the KAN design

To isolate the contribution of the KAN module, we constructed an MLP-based variant of DFCE-KanT by substituting all KAN blocks with conventional multi-layer perceptrons, while keeping the rest of the architecture unchanged.

Correlation performance (HEG and HVG, ↑): The full DFCE-KanT consistently outperforms the MLP variant across all datasets. For example, on the HBC dataset, DFCE-KanT improves HEG from 0.3004 to 0.4051 (+35%) and HVG from 0.1819 to 0.2294 (+26%). Similar improvements are observed across all other datasets, including a +13% increase in HEG on cSCC (0.3909 vs. 0.3450) and a +37% increase on Liver (0.2035 vs. 0.1490). These results demonstrate that the KAN module is more effective at capturing the complex nonlinear relationships present in gene expression data.

Prediction error (MSE and MAE, ↓): DFCE-KanT also achieves lower prediction errors on most datasets. Specifically, it obtains the lowest MSE and MAE on cSCC, Alex, and HBC. Although the MLP variant shows slightly lower error values on HER2+ and Liver, its correlation performance is substantially weaker, indicating that

the improvements are achieved by overfitting simple distributional properties rather than capturing meaningful expression patterns.

b. Effectiveness of Contrastive Learning

We compared our full model to a direct regression baseline with the same network, using MSE loss instead of contrastive loss.

Correlation performance (HEG and HVG, ↑): The direct regression variant exhibits poor correlation performance across all datasets. Notably, on the cSCC dataset, it yields near-zero correlation (HEG = 0.0137, HVG = 0.0119), indicating a complete failure to learn meaningful expression patterns. In contrast, DFCE-KanT achieves strong correlation (0.3909 and 0.2962), demonstrating that the contrastive objective enables the model to learn discriminative and robust representations.

Prediction error (MSE and MAE, ↓): While the regression variant occasionally achieves low error values (e.g., MSE = 0.2902, MAE = 0.4022 on HER2+), these come at the cost of extremely poor correlation, reflecting a trivial fit to the data distribution rather than predictive capability. On the other hand, DFCE-KanT achieves the lowest error on the majority of datasets (cSCC, Alex, HBC, Liver) while maintaining significantly higher correlation, confirming that contrastive learning leads to both more accurate and more biologically meaningful predictions.

Control experiments confirm the KAN module and contrastive learning are essential. KAN outperforms MLPs for nonlinear gene expression modeling. Contrastive learning extracts stronger features than direct regression, improving prediction performance across all datasets.

2 Supplementary Figures

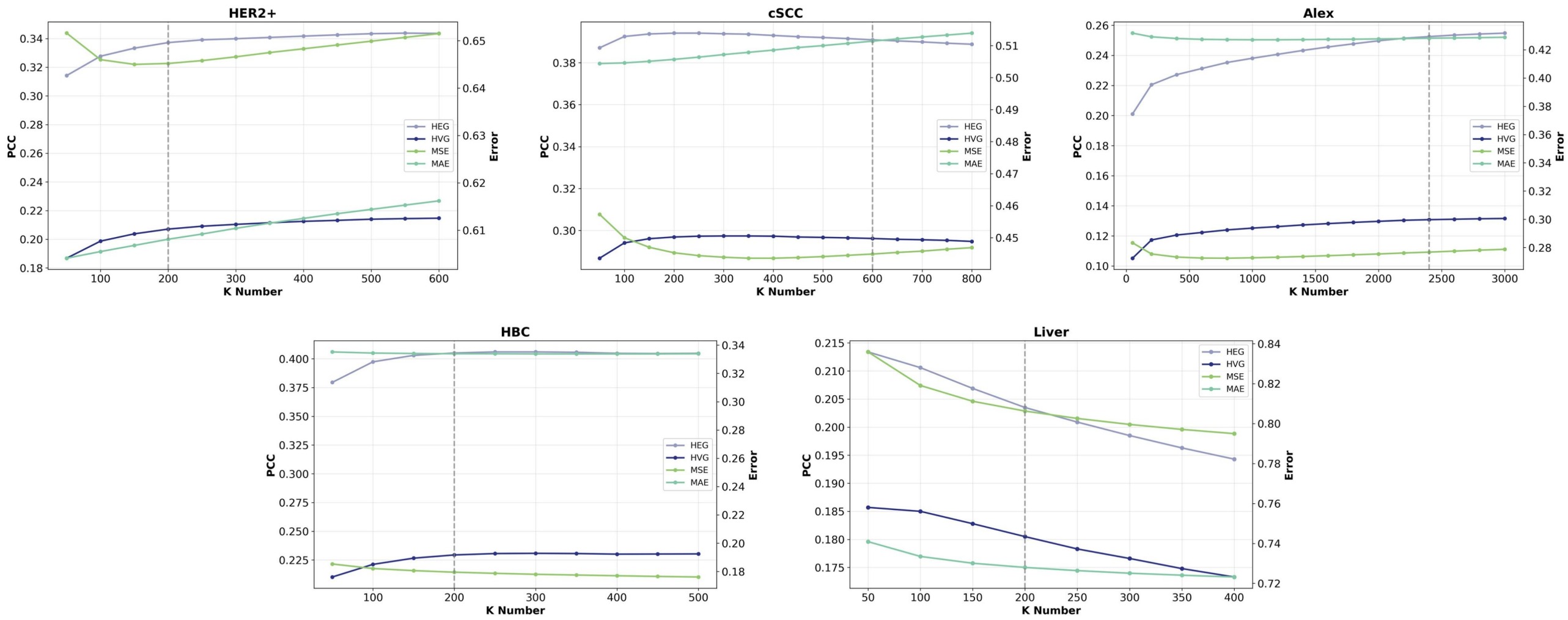

Fig. S1. Impact of K values on prediction performance and identification of the optimal K for HER2+, cSCC, Alex, HBC and Liver datasets.

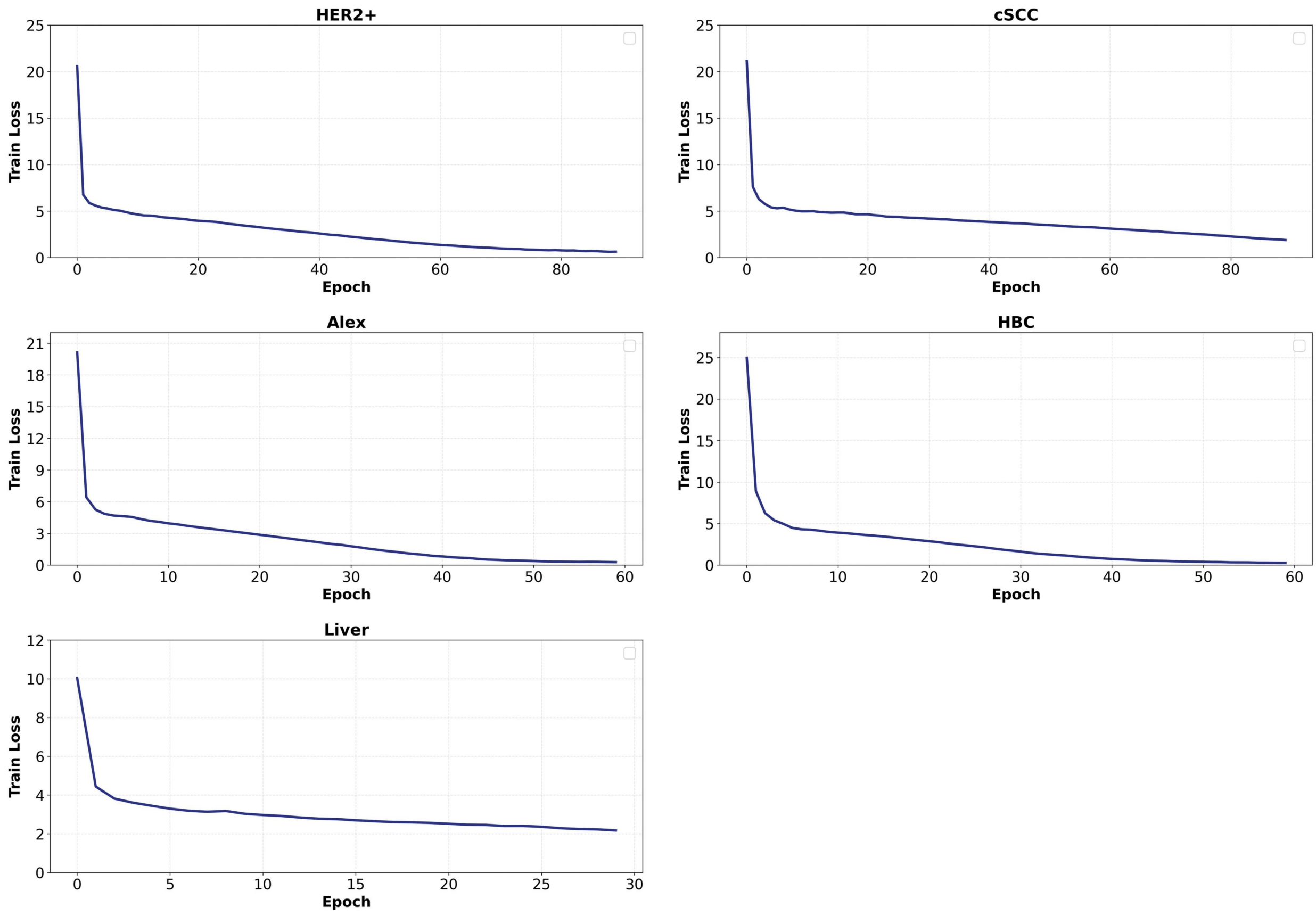

Fig. S2. Training loss curves and epoch settings across HER2+, cSCC, Alex, HBC and Liver datasets.

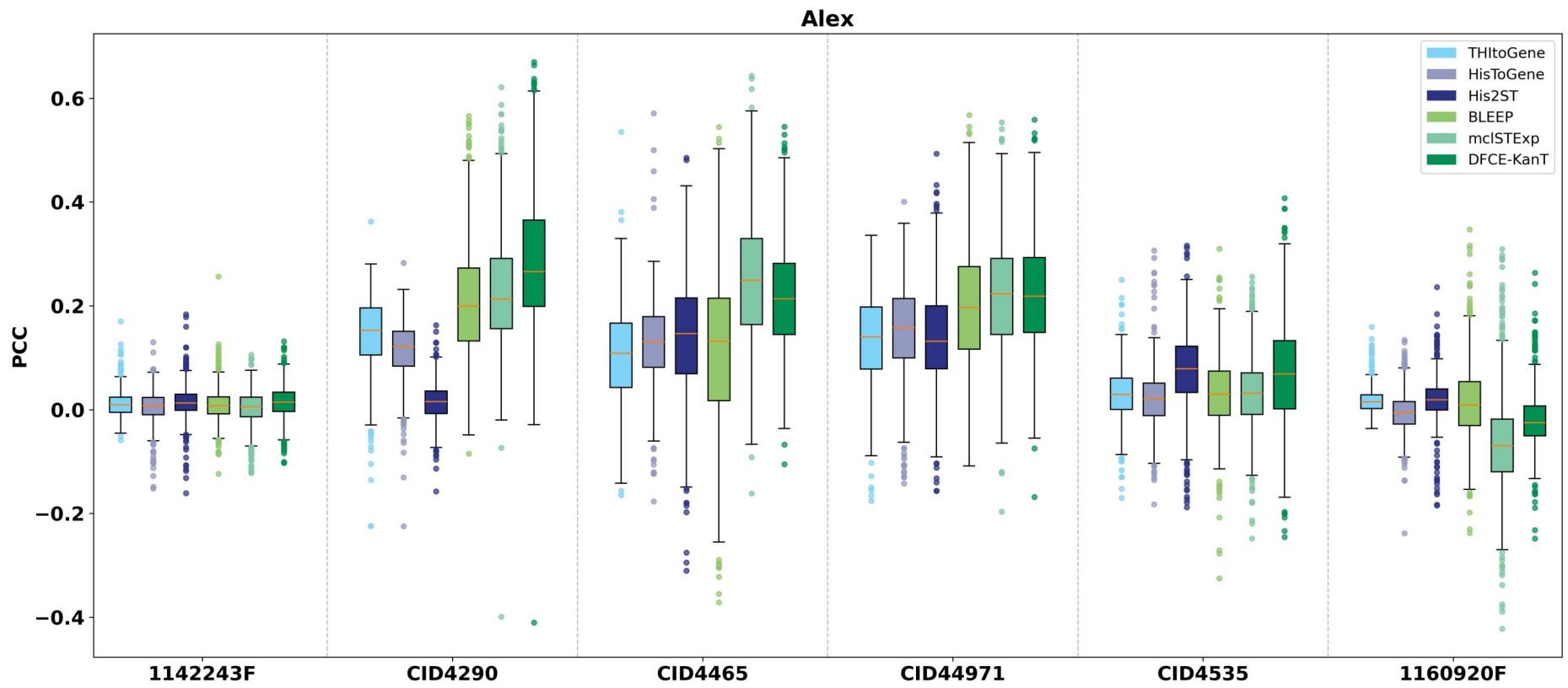

Fig. S3. Section-wise performance comparison on the Alex dataset. PCC was calculated to evaluate the correlation between observed and predicted gene expression across six models: THItoGene, HisToGene, His2ST, BLEEP, mclSTExp and DFCE-KanT.

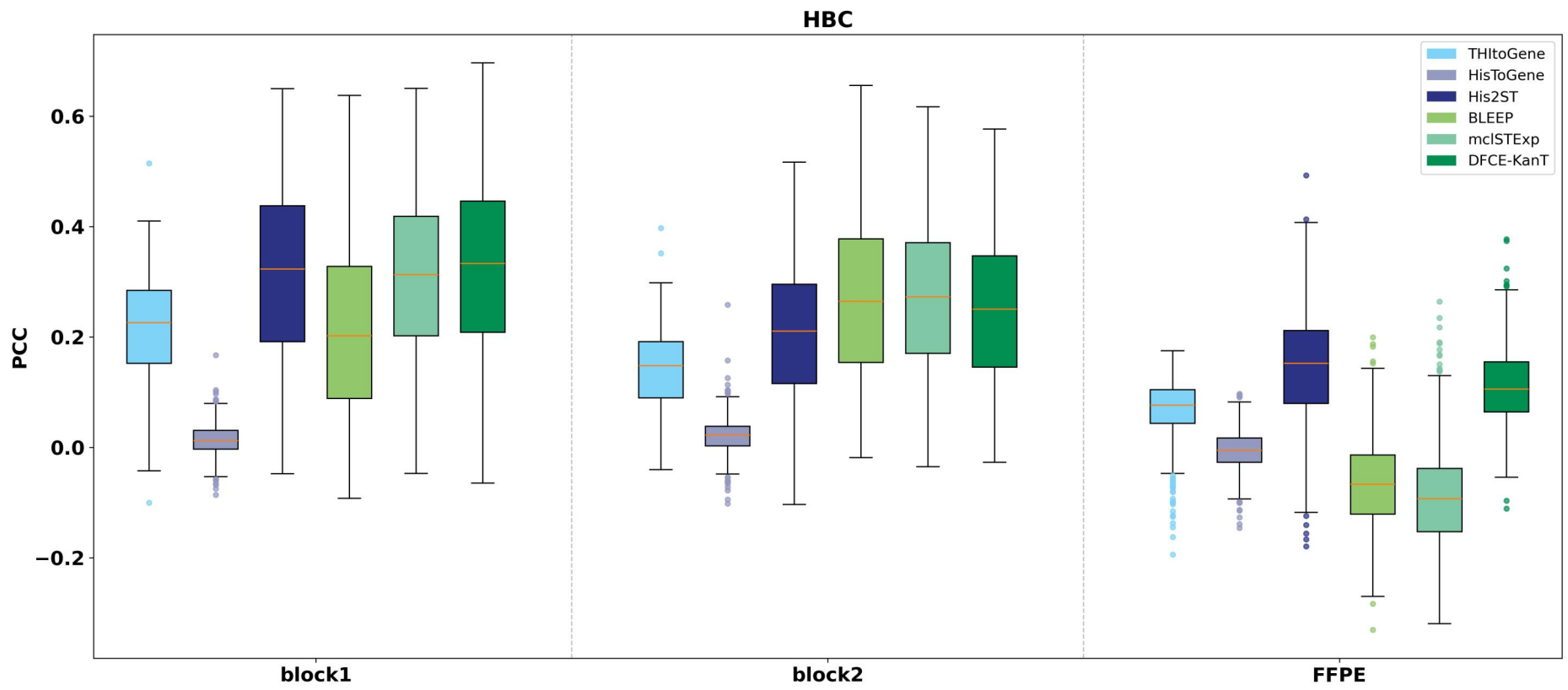

Fig. S4. Section-wise performance comparison on the HBC dataset. PCC was calculated to evaluate the correlation between observed and predicted gene expression across six models: THItoGene, HisToGene, His2ST, BLEEP, mclSTExp and DFCE-KanT.

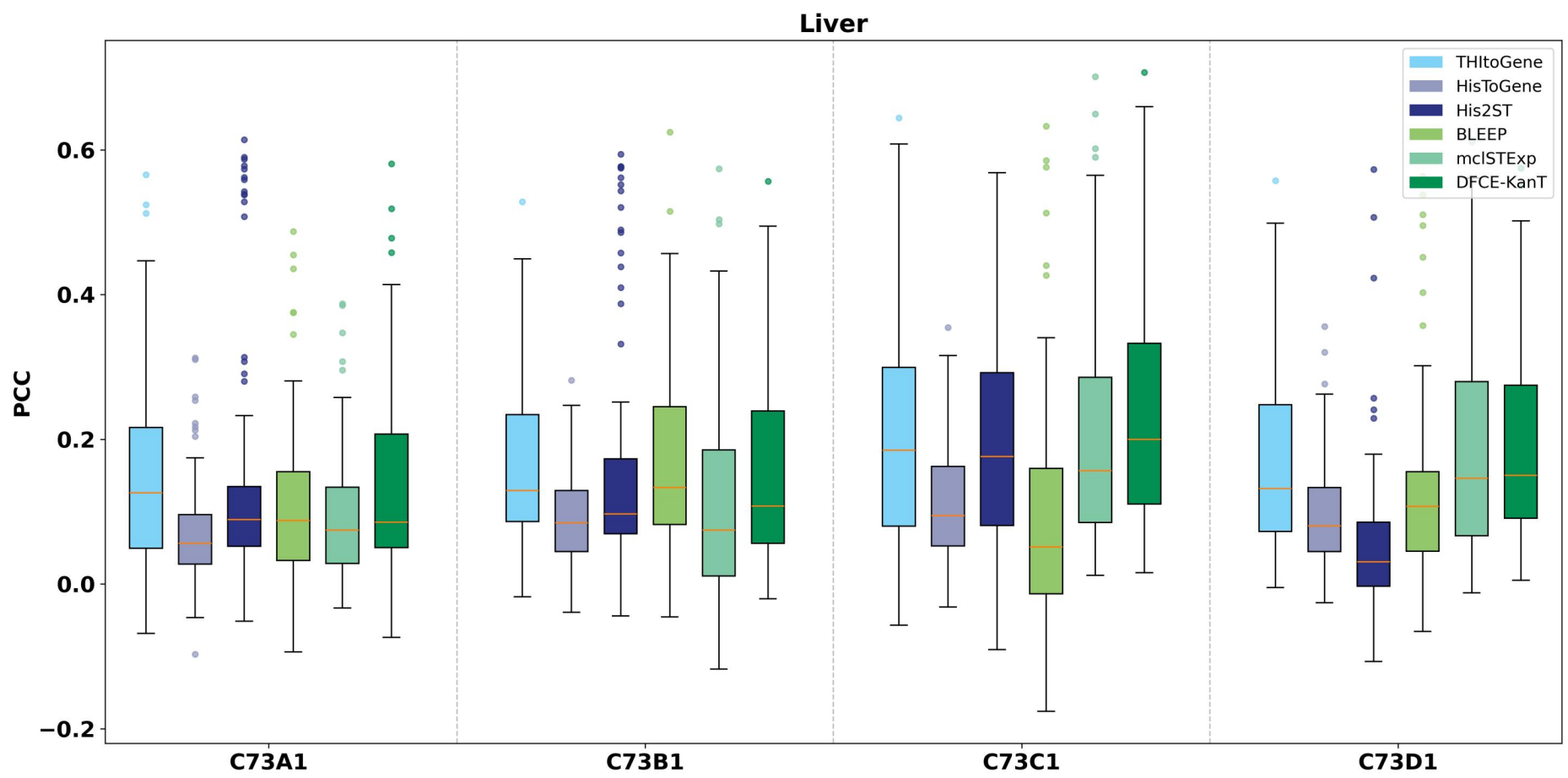

Fig. S5. Section-wise performance comparison on the Liver dataset. PCC was calculated to evaluate the correlation between observed and predicted gene expression across six models: THItoGene, HisToGene, His2ST, BLEEP, mclSTExp and DFCE-KanT.

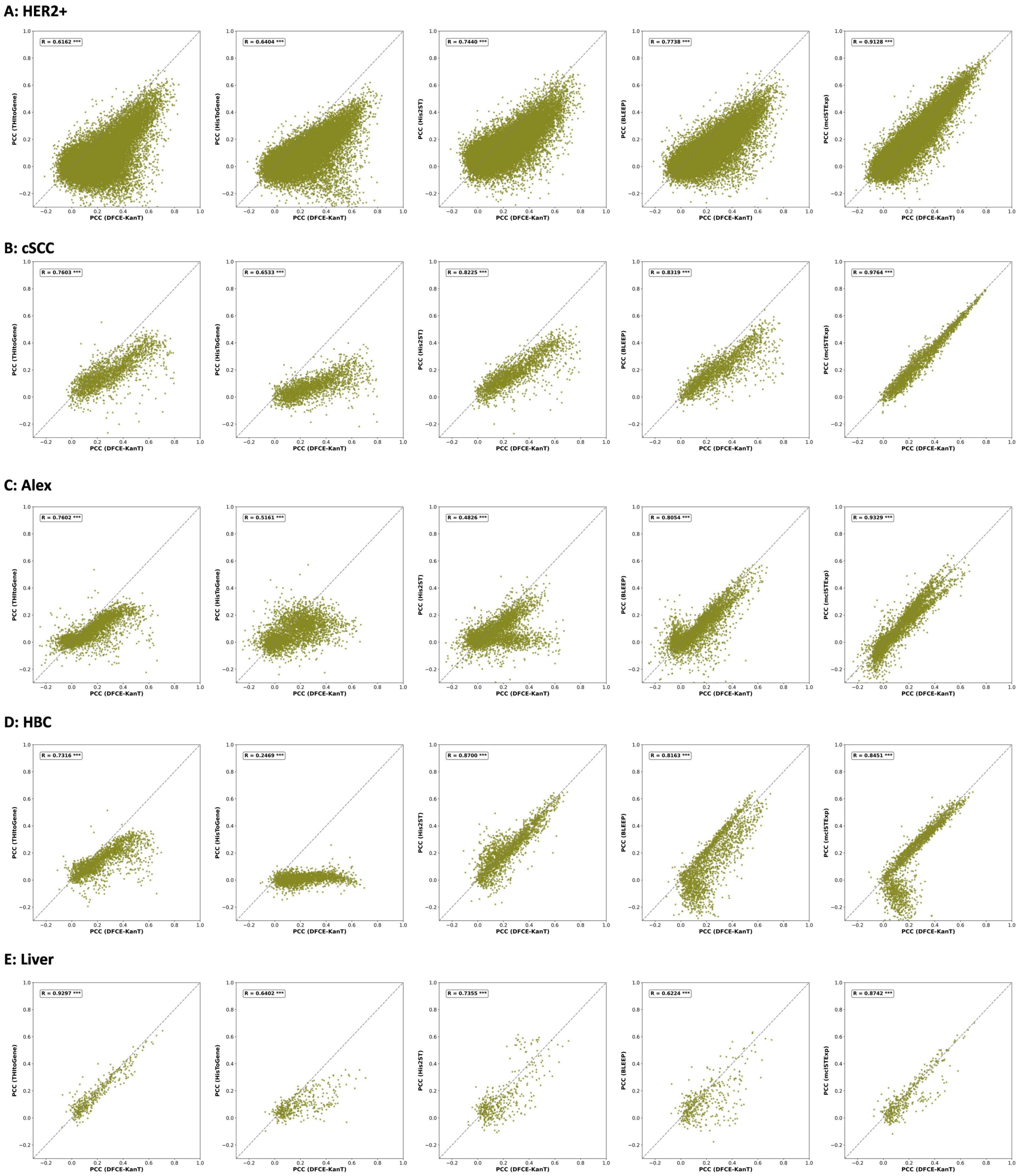

Fig. S6. Pairwise PCC correlation among six models on five datasets. Correlations between DFCE-KanT and THItoGene, HisToGene, His2ST, BLEEP and mclSTExp were assessed using PCC values obtained from HER2+, cSCC, Alex, HBC and Liver datasets.

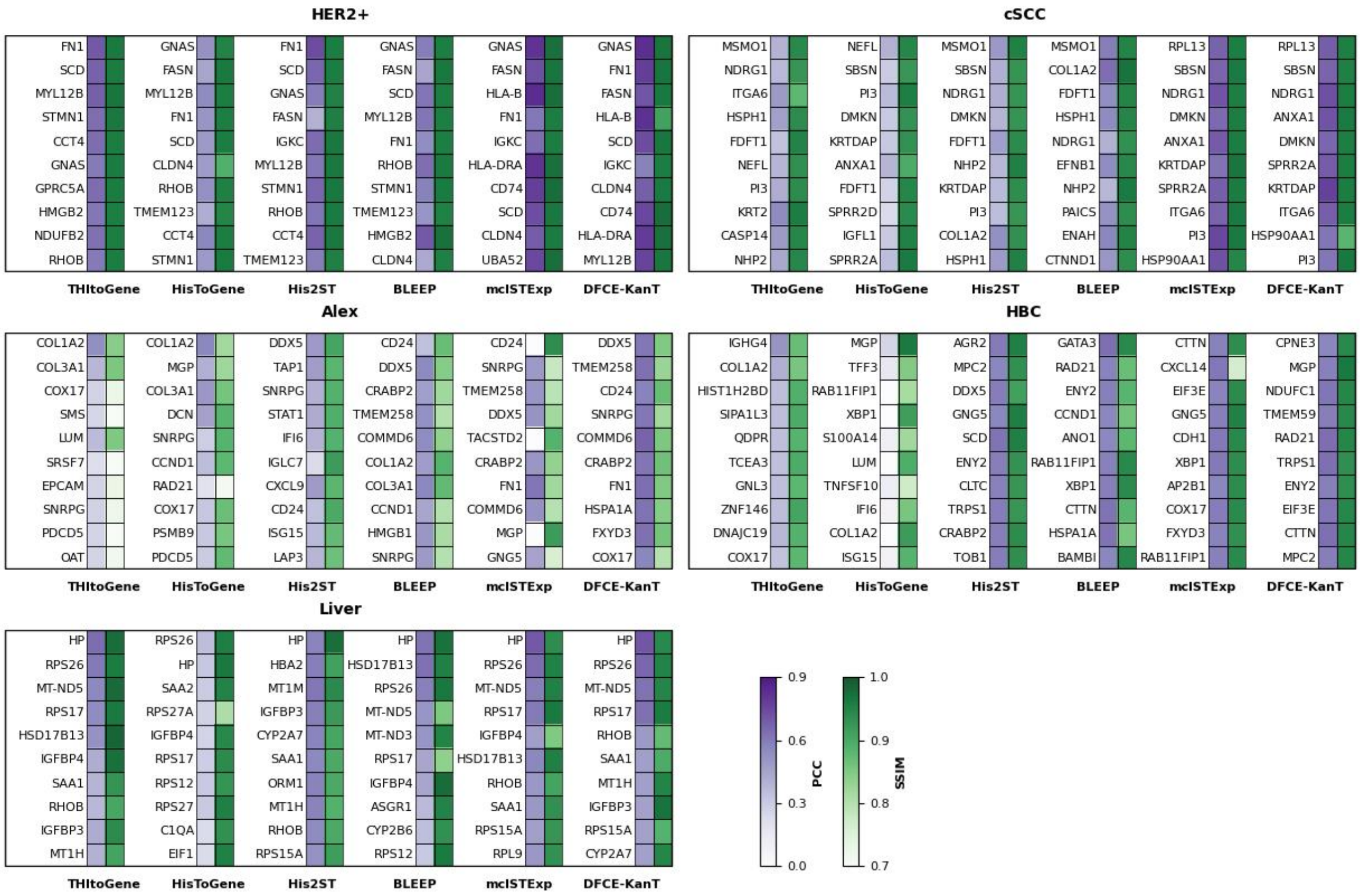

Fig. S7. PCC and SSIM of the top 10 predicted genes by THItoGene, HisToGene, His2ST, BLEEP, mclSTExp and DFCE-KanT across HER2+, cSCC, Alex, HBC and Liver datasets.

3 Supplementary Tables

Table. S1. The PCC per tissue section on the HER2+ dataset

Sample	THIToGene	HisToGene	His2ST	BLEEP	mclSTExp	DFCE-KanT
A2	0.0335	0.0329	0.0815	0.0524	0.0471	0.0948
A3	0.0208	0.0356	0.0508	0.0636	0.1174	0.1170
A4	-0.0236	0.0333	0.0337	0.0317	0.0617	0.0875
A5	0.0456	0.0201	0.0746	0.0844	0.0630	0.0862
A6	0.0058	0.0283	0.0984	0.0581	-0.0008	0.0695
B1	0.2666	0.1714	0.2931	0.2196	0.3897	0.4068
B2	0.2981	0.1930	0.3239	0.2887	0.4610	0.4205
B3	0.2480	0.1717	0.2809	0.2608	0.3676	0.4194
B4	0.2787	0.2110	0.3052	0.3046	0.4666	0.4650
B5	0.2026	0.1633	0.2324	0.2169	0.3504	0.3808
B6	0.2643	0.1868	0.2922	0.2389	0.3855	0.3904
C1	0.0543	0.1052	0.1710	0.2040	0.1778	0.3080
C2	0.0119	0.0969	0.1184	0.1684	0.2883	0.2851
C3	0.0735	0.0796	0.1856	0.0647	0.2559	0.2943
C4	0.0892	0.0945	0.1676	0.1845	0.2496	0.2837
C5	0.0724	0.0774	0.0932	0.1766	0.1885	0.2493
C6	0.0130	0.1015	0.1407	0.0682	0.2324	0.1747
D1	0.0506	0.0779	0.1488	0.0943	0.1831	0.2252
D2	0.0953	0.0956	0.1530	0.0959	0.2150	0.1926
D3	0.0993	0.0929	0.1694	0.1089	0.1934	0.2281
D4	0.0185	0.1162	0.2000	0.1087	0.2330	0.2402
D5	-0.0137	0.0937	0.1352	0.1118	0.2158	0.2379
D6	0.0091	0.0970	0.1113	0.0556	0.1917	0.1974
E1	0.0150	0.0017	0.0824	0.0337	-0.0081	0.0323
E2	0.0162	0.0019	0.0872	0.0242	0.0267	0.0339
E3	-0.0054	-0.0024	0.0923	0.0372	0.0228	0.0573
F1	-0.0025	-0.0013	0.0695	0.0268	0.0121	0.0017
F2	0.0006	0.0041	0.0721	0.0185	0.0518	0.0249
F3	0.0099	-0.0050	0.0430	0.0143	0.0347	0.0211
G1	-0.0088	0.0902	0.1158	0.1270	0.1469	0.1959
G2	0.0069	0.0973	0.0889	0.1178	0.1658	0.2117
G3	0.0439	0.1010	0.1051	0.1222	0.1756	0.1939

Table. S2. The PCC per tissue section on the cSCC dataset

Sample	THIToGene	HisToGene	His2ST	BLEEP	mclSTExp	DFCE-KanT
P2_ST_rep1	0.3128	0.1367	0.2828	0.2702	0.4410	0.4647
P2_ST_rep2	0.2661	0.0845	0.2778	0.3678	0.4973	0.5226
P2_ST_rep3	0.3102	0.1603	0.3353	0.3685	0.4669	0.4962
P5_ST_rep1	0.0858	0.0123	0.0765	0.0819	0.1182	0.1553
P5_ST_rep2	0.1193	0.0212	0.1105	0.0814	0.1008	0.1242
P5_ST_rep3	0.1191	0.0149	0.0660	0.0673	0.0922	0.1100
P9_ST_rep1	0.1816	0.1148	0.1925	0.1892	0.3265	0.3931
P9_ST_rep2	0.2081	0.1182	0.2195	0.2310	0.3233	0.3632
P9_ST_rep3	0.1295	0.0866	0.1789	0.1992	0.2292	0.2675
P10_ST_rep1	0.1621	0.0616	0.1613	0.1618	0.2541	0.2266
P10_ST_rep2	0.1231	0.0418	0.1302	0.2054	0.2464	0.2471
P10_ST_rep3	0.1304	0.0693	0.1533	0.1691	0.1532	0.1840

Table. S3. The PCC per tissue section on the Alex dataset

Sample	THIToGene	HisToGene	His2ST	BLEEP	mclSTExp	DFCE-KanT
1142243F	0.0110	0.0051	0.0137	0.0092	0.0029	0.0148
CID4290	0.1438	0.1129	0.0142	0.2101	0.2264	0.2804
CID4465	0.1028	0.1275	0.1379	0.1163	0.2469	0.2155
CID44971	0.1341	0.1505	0.1378	0.1997	0.2234	0.2218
CID4535	0.0295	0.0219	0.0755	0.0328	0.0335	0.0711
1160920F	0.0169	-0.0049	0.0189	0.0140	-0.0671	-0.0188

Table. S4. The PCC per tissue section on the HBC dataset

Sample	THIToGene	HisToGene	His2ST	BLEEP	mclSTExp	DFCE-KanT
block1	0.2107	0.0127	0.3098	0.2174	0.3092	0.3266
block2	0.1389	0.0208	0.2034	0.2699	0.2722	0.2500
FFPE	0.0675	-0.0052	0.1463	-0.0678	-0.0925	0.1117

Table. S5. The PCC per tissue section on the Liver dataset

Sample	THIToGene	HisToGene	His2ST	BLEEP	mclSTExp	DFCE-KanT
C73A1	0.1526	0.0706	0.1410	0.1054	0.0911	0.1380
C73B1	0.1707	0.0966	0.1607	0.1692	0.1255	0.1615
C73C1	0.2007	0.1128	0.1938	0.0939	0.2019	0.2372
C73D1	0.1628	0.0956	0.0551	0.1233	0.1821	0.1853

Table S6.The top 10 predicted genes with the highest mean −log10(P-value) on the cSCC dataset

Gene	PCC						SSIM					
	THIToGene	HisToGene	His2ST	BLEEP	mclSTExp	DFCE-KanT	THIToGene	HisToGene	His2ST	BLEEP	mclSTExp	DFCE-KanT
RPL13	0.3813	0.2306	0.2449	0.2142	0.6708	0.6859	0.9375	0.9309	0.9343	0.9185	0.9568	0.9556
SBSN	0.3692	0.3033	0.3372	0.3597	0.6631	0.6660	0.9290	0.9253	0.9325	0.9322	0.9618	0.9548
NDRG1	0.3743	0.2225	0.3177	0.3670	0.7164	0.7295	0.9265	0.9278	0.9308	0.9310	0.9576	0.9579
ANXA1	0.2471	0.2038	0.2071	0.1759	0.6976	0.7162	0.9275	0.9170	0.9182	0.9333	0.9597	0.9615
DMKN	0.4124	0.3031	0.3788	0.3059	0.6527	0.6585	0.9380	0.9346	0.9405	0.9378	0.9593	0.9578
SPRR2A	0.2316	0.1734	0.1329	0.1472	0.6804	0.6959	0.9129	0.9345	0.9127	0.9292	0.9602	0.9592
KRTDAP	0.1381	-0.1264	0.3330	0.2897	0.7646	0.7703	0.9521	0.9326	0.9464	0.9653	0.9618	0.9598
ITGA6	0.2937	0.1957	0.2496	0.2640	0.6657	0.6849	0.9267	0.9348	0.9210	0.9259	0.9628	0.9590
HSP90AA1	0.1931	0.1045	0.1850	0.1205	0.5969	0.6218	0.9219	0.9531	0.9142	0.9339	0.9582	0.8835
PI3	0.3205	0.2517	0.2871	0.3385	0.6103	0.6134	0.9328	0.9290	0.9388	0.9349	0.9643	0.9648

Table S7.The top 10 predicted genes with the highest mean −log10(P-value) on the Alex dataset

Gene	PCC						SSIM					
	THIToGene	HisToGene	His2ST	BLEEP	mclSTExp	DFCE-KanT	THIToGene	HisToGene	His2ST	BLEEP	mclSTExp	DFCE-KanT
DDX5	0.1063	0.0660	0.0427	0.5575	0.5178	0.6122	0.7251	0.8043	0.8126	0.8434	0.8194	0.8493
TMEM258	0.2305	0.1605	0.0273	0.5274	0.5113	0.6316	0.7287	0.7865	0.7820	0.8008	0.7930	0.8268
CD24	-0.2240	-0.2250	0.0012	0.4479	0.4947	0.5796	0.8214	0.8049	0.8318	0.8187	0.8357	0.8709
SNRPG	0.2711	0.1892	0.1014	0.4891	0.4914	0.6139	0.7216	0.7873	0.7802	0.7996	0.7807	0.8187
COMMD6	0.1987	0.1093	0.0829	0.5517	0.5230	0.6700	0.7196	0.8178	0.8050	0.8347	0.7993	0.8522
CRABP2	0.0230	-0.0278	-0.0061	0.4724	0.5043	0.6183	0.7807	0.7955	0.8094	0.8207	0.8325	0.8637
FN1	-0.0234	0.1149	-0.0565	0.4477	0.6216	0.6376	0.8038	0.8399	0.8361	0.8605	0.8213	0.8491
HSPA1A	0.2072	0.1228	-0.0053	0.4534	0.4994	0.6253	0.7721	0.7803	0.8028	0.8208	0.8027	0.8439
FXYD3	-0.0080	-0.0034	-0.0873	0.4421	0.4934	0.6309	0.8173	0.8051	0.7993	0.7869	0.8406	0.8582
COX17	0.2692	0.1821	0.0428	0.4371	0.4250	0.5675	0.7258	0.7518	0.7678	0.7903	0.7605	0.7988

Table S8.The top 10 predicted genes with the highest mean −log10(P-value) on the HBC dataset

	PCC						SSIM					
Gene	THIToGene	HisToGene	His2ST	BLEEP	mclSTExp	DFCE-KanT	THIToGene	HisToGene	His2ST	BLEEP	mclSTExp	DFCE-KanT
CPNE3	0.2700	-0.0268	0.5457	0.4480	0.5467	0.5720	0.8649	0.8950	0.9334	0.9381	0.9347	0.9423
MGP	0.2363	0.1670	0.2010	0.5267	0.5102	0.5845	0.8535	0.8867	0.9432	0.9594	0.9576	0.9628
NDUFC1	0.3599	-0.0191	0.5355	0.5182	0.5877	0.6166	0.8613	0.8911	0.9317	0.9257	0.9407	0.9465
TMEM59	0.2904	-0.0065	0.5150	0.5134	0.5636	0.5962	0.6685	0.8750	0.9244	0.9434	0.9381	0.9442
RAD21	0.2772	-0.0243	0.6066	0.5379	0.5904	0.6389	0.8710	0.9345	0.9370	0.9414	0.9372	0.9461
TRPS1	0.2233	-0.0281	0.6002	0.5644	0.6148	0.6356	0.8652	0.9379	0.9254	0.9295	0.9308	0.9351
ENY2	0.3237	-0.0258	0.5974	0.5212	0.5592	0.5883	0.8776	0.9141	0.9306	0.9329	0.9303	0.9357
EIF3E	0.2606	-0.0416	0.5612	0.5063	0.5756	0.6167	0.6633	0.8936	0.9392	0.9436	0.9396	0.9457
CTTN	0.2076	-0.0091	0.6250	0.4694	0.5819	0.6346	0.8926	0.9455	0.9389	0.9237	0.9369	0.9463
MPC2	0.2690	-0.0064	0.5726	0.5061	0.5449	0.5956	0.8578	0.9324	0.9330	0.9409	0.9385	0.9464

Table S9.The top 10 predicted genes with the highest mean −log10(P-value) on the Liver dataset

	PCC						SSIM					
Gene	THIToGene	HisToGene	His2ST	BLEEP	mclSTExp	DFCE-KanT	THIToGene	HisToGene	His2ST	BLEEP	mclSTExp	DFCE-KanT
HP	0.6442	0.2955	0.5683	0.5762	0.7013	0.7073	0.9759	0.9541	0.9764	0.9677	0.9367	0.9437
RPS26	0.6080	0.3546	0.5285	0.5854	0.6498	0.6597	0.9610	0.9284	0.8351	0.9656	0.9524	0.9515
MT-ND5	0.5600	0.2185	0.5415	0.2981	0.5901	0.6230	0.9799	0.9547	0.9723	0.9696	0.9531	0.9478
RPS17	0.5470	0.2894	0.4119	0.4268	0.6019	0.6323	0.9627	0.9395	0.9376	0.9560	0.9636	0.9606
RHOB	0.3409	0.1081	0.4606	0.0998	0.2637	0.4905	0.9340	0.8549	0.9143	0.9755	0.7914	0.8731
SAA1	0.3164	0.1058	0.4578	0.0930	0.2588	0.4724	0.9587	0.8991	0.9249	0.9732	0.9175	0.8959
MT1H	0.3999	0.1980	0.5774	0.2740	0.4241	0.4714	0.9063	0.9373	0.8895	0.9249	0.9050	0.9495
IGFBP3	0.4206	0.2081	0.5766	0.2862	0.4207	0.4590	0.9392	0.9502	0.9223	0.9326	0.9245	0.9702
RPS15A	0.3263	0.0852	0.4973	0.1359	0.2825	0.4626	0.9290	0.8698	0.9121	0.9773	0.9203	0.8874
CYP2A7	0.3861	0.1842	0.5617	0.2804	0.4262	0.4644	0.8943	0.9021	0.8900	0.9215	0.8977	0.9465

Table. S10. Performance of DFCE-KanT and its ablation variants on five datasets. We assess the efficacy of the KanT module and FCE attention by replacing them with MLP, Transformer, SE and CBAM. Metrics include HEG, HVG, MSE and MAE. Bold values indicate the best performance.

Methods	HER2+		cSCC		Alex		HBC		Liver	
	HEG(↑)	HVG(↑)	HEG(↑)	HVG(↑)	HEG(↑)	HVG(↑)	HEG(↑)	HVG(↑)	HEG(↑)	HVG(↑)
+MLP	0.3135	0.1889	0.3486	0.2604	0.1957	0.1104	0.3080	0.1740	0.1285	0.1214
+Transformer	0.3308	0.2043	0.3831	0.2904	0.2023	0.1165	0.2322	0.1212	0.1604	0.1514
+SE	0.3263	0.2044	0.3359	0.2557	0.2035	0.1163	0.3249	0.1890	0.1543	0.1465
+CBAM	0.3181	0.1893	0.3503	0.2655	0.1829	0.1094	0.2239	0.0913	0.1670	0.1503
**DFCE-KanT**	**0.3372**	**0.2071**	**0.3909**	**0.2962**	**0.2525**	**0.1308**	**0.4051**	**0.2294**	**0.2035**	**0.1805**
	MSE	MAE	MSE(↓)	MAE(↓)	MSE(↓)	MAE(↓)	MSE(↓)	MAE(↓)	MSE	MAE
+MLP	0.6290	0.5945	0.4636	0.5377	0.2745	0.4411	0.1866	0.3391	0.7373	0.6913
+Transformer	**0.6367**	0.6099	0.4456	0.5203	0.2788	0.4402	0.1806	0.3400	1.0967	0.8772
+SE	0.6445	0.6143	0.4608	0.5282	0.2835	0.4493	0.1891	0.3417	0.9541	0.8106
+CBAM	0.6497	**0.6061**	0.4566	0.5370	**0.2761**	0.4382	0.1878	0.3537	**0.7133**	**0.6824**
**DFCE-KanT**	0.6452	0.6081	**0.4449**	**0.5114**	0.2768	**0.4282**	**0.1795**	**0.3339**	0.8063	0.7280

Table. S11. Comparison between DFCE-KanT and two control baselines on five spatial transcriptomics datasets. We verify the effectiveness of KAN blocks and contrastive learning via two variants: MLP substitution and direct regression. All models are evaluated using HEG, HVG, MSE and MAE. Bold values indicate the best performance.

Methods	HER2+		cSCC		Alex		HBC		Liver	
	HEG(↑)	HVG(↑)	HEG(↑)	HVG(↑)	HEG(↑)	HVG(↑)	HEG(↑)	HVG(↑)	HEG(↑)	HVG(↑)
MLP	0.3056	0.1846	0.3450	0.2583	0.2005	0.1104	0.3004	0.1819	0.1490	0.1365
Regression	0.1336	0.1146	0.0137	0.0119	0.1887	0.0993	0.3052	0.2017	0.1588	0.1452
**DFCE-KanT**	**0.3372**	**0.2071**	**0.3909**	**0.2962**	**0.2525**	**0.1308**	**0.4051**	**0.2294**	**0.2035**	**0.1805**
	MSE	MAE	MSE(↓)	MAE(↓)	MSE(↓)	MAE(↓)	MSE(↓)	MAE(↓)	MSE	MAE
MLP	0.6314	0.5885	0.4664	0.5368	0.2785	0.4494	0.1942	0.3451	**0.7099**	**0.6776**
Regression	**0.2902**	**0.4022**	0.8072	0.6198	0.3049	0.4421	0.1757	0.3389	0.8823	0.7719
**DFCE-KanT**	0.6452	0.6081	**0.4449**	**0.5114**	**0.2768**	**0.4282**	0.1795	**0.3339**	0.8063	0.7280

Table. S12. Summary of the datasets.

Dataset	Tissue	WSIs
HER2+	human HER2-positive breast tumor	32
cSCC	human cutaneous squamous cell carcinoma	12
Alex	human breast cancer	6
HBC	human breast cancer	3
Liver	human liver	4
